# Impact of food availability on the thermal performance curves of male European green lizards (*Lacerta viridis*)

**DOI:** 10.1007/s00442-025-05699-z

**Published:** 2025-04-01

**Authors:** Boglárka Mészáros, Lilla Jordán, Orsolya Molnár, János Török

**Affiliations:** 1 HUN-REN Balaton Limnological Research Institute, Klebelsberg Kuno Street 3., H-8237 Tihany, Hungary; 2 Behavioural Ecology Group, Department of Systematic Zoology and Ecology, ELTE Eötvös Loránd University, Pázmány Péter Sétány 1/C, 1117 Budapest, Hungary; 3 Leibniz Institute for Zoo and Wildlife Research, Alfred-Kowalke-Str. 17, 10315 Berlin, Germany; 4 HUN-REN-ELTE-MTM Integrative Ecology Research Group, Pázmány Péter Sétány 1/C, 1117 Budapest, Hungary

**Keywords:** Changing environment, Food availability, Locomotor performance, Lacertidae

## Abstract

In a changing environment, characterized by human-induced rapid environmental change (HIREC), understanding the impacts of environmental stressors on reptile species is crucial. Preferred body temperatures (PBT) and thermal performance curves (TPCs) are comprehensive thermal physiology traits reflecting overall physiological performance and crucial for predicting species-specific responses to environmental changes. There is limited or conflicting information on how food availability affect the PBT and TPCs of lizard species, despite their significance in the context of the global decline in reptile species. The aim of this study was to experimentally investigate how food deprivation affects the PBT and TPCs of the European green lizards (*Lacerta viridis*). We exposed 30 adult male European green lizards to optimal and suboptimal food treatments. We assessed PBTs, and characterized the TPCs based on the thermal optimum (T_o_), the maximum performance (P_max_) and performance breadth (B_80_) of the lizards. We found that food availability had a significant impact on preferred body temperature and locomotor performance. Lizards experiencing suboptimal conditions showed a preference for lower body temperatures, indicating an intention to minimize energy expenditure during fasting. Additionally, food-deprived lizards had wider B_80_ range, suggesting their thermal acclimatization to maintain effective performance across a broader temperature range. Our findings highlight the importance of food availability as a key environmental stressor influencing thermoregulation strategies. As habitat modifications and global warming continue, it is crucial to evaluate the impacts of these changes on species for the development of effective conservation strategies.

**Supplementary Information:**

The online version contains supplementary material available at 10.1007/s00442-025-05699-z.

## Introduction

Ectothermic organisms perform behavioral thermoregulation by adjusting their body temperature through positional changes between cooler and warmer microhabitats (Giacometti et al. 2021; May 1979). This behavior is crucial, as body temperature is the most important ecophysiological factor influencing various aspects of their behavior and physiology (Seebacher 2005), such as immune function (Ding et al. 2024; Sacchi et al. 2014), metabolic rate (Giacometti et al. 2022) and can substantially affect locomotor performance (Araspin et al. 2023; Cabezas-Cartes et al. 2023; Nisani et al. 2022; Ovallez et al. 2023).

The efficiency of an organism’s locomotor performance serves as a comprehensive trait reflecting the organism’s overall abilities. Its importance is emphasized by its strong link to fitness-related traits like foraging, mating, and avoiding predators (Angilletta et al. 2002b; Irschick et al. 2008). Determining the thermal dependence of locomotor performance is important as it provides valuable insights into a species’ vulnerability and sensitivity to temperature changes. These insights are essential for predicting how ectotherm species will respond to environmental changes, as factors influencing survival, reproductive success, and overall fitness are interlinked (Sinervo et al. 2010). Numerous studies have investigated the impact of temperature fluctuations on the locomotor performance of ectotherm species (Clusella-Trullas et al. 2011; Clusella-Trullas and Chown 2014; Araspin et al. 2023; Cabezas-Cartes et al. 2023), primarily focusing on temperature acclimatization while giving less attention to other important factors such as water availability, social interactions, or diet.

Human-induced rapid environmental change (HIREC), such as habitat degradation, fragmentation, urbanization or climate change, not only modifies thermal habitats but also influences food resources, leading to fluctuations in prey abundance (Huey and Kingsolver 2019; Van der Putten et al. 2010). Furthermore, shifts in thermal habitats may restrict the available time for foraging and mating activities in ectotherms (Huey and Kingsolver 2019; Sinervo et al. 2010). These environmental stressors, encompassing changes in food availability have the potential to reduce survivorship and reproductive output, ultimately contributing to the decline of reptile species (Gibbons et al. 2000).

Studies investigating the consequences of temporary starvation on body temperatures and locomotor performance of reptiles found conflicting results. For instance, research on Ching Hai toadhead agama (*Phrynocephalus vlangalii*) demonstrated that food availability directly influences tail display intensity, suggesting that lizards trade off their display intensity according to food accessibility (Zhu et al. 2020). Besides this, a study on the ornate tree lizards (*Urosaurus ornatus*) showed that starving males had reduced stamina after a four-week long food limitation experiment (Jaworski and Lattanzio 2017). Contrary to these findings, Zhihua and Xiang (2005) discovered that the locomotor performance of Chinese blue-tailed skinks (*Plestiodon chinensis*) significantly increased after a three-day period of food deprivation, compared to individuals maintained under optimal food conditions. In a long-term (40-day) experiment, Shu et al. (2010) investigated the same species and found that the locomotor performance of individuals was influenced by thermoregulation time but not by the quantity of food. Additionally, Lailvaux et al. (2020) observed that dietary restriction had no impact on sprint speed in green anole (*Anolis carolinensis*) lizards when combined with training despite notable reductions in body mass.

In these previous studies, locomotor performance was characterized by tail display intensity, endurance and maximum running speed. However, the relationship between body temperature and performance was not explored. The temperature-dependence of specific performance aspects, such as locomotor performance, is commonly described using thermal performance curves (TPCs). The association is illustrated through an asymmetric function, where peak performance is observed at an intermediate temperature (Angilletta et al. 2002a, 2002b). TPCs can be characterized with various variables, offering insights into the connection between the thermal environment and performance. These variables include the peak performance (maximum performance), the thermal optimum, the effective performance (performance breadth) and the critical thermal limits. Maximum performance (P_max_) indicates the peak performance on the TPCs, while the thermal optimum (T_o_) represents the temperature point at which performance reaches its maximum. Performance breadth (B_80_) signifies the temperature range enabling performance at or above a specified threshold, such as 80%. Critical thermal limits establish the lowest (CT_min_) and the highest (CT_max_) body temperatures at which performance can still occur (Angilletta et al. 2002b; Bulté and Blouin-Demers 2006). In addition to TPCs, the preferred body temperature (PBT) is a crucial element of physiological performance. Thermoregulating ectotherms usually seek to maintain their internal temperature at a PBT to enhance essential processes, such as digestion and locomotion. Coevolving with the T_o_, the PBT is highly interlinked with the shape of thermal performance curves (Angilletta et al. 2002a, 2002b).

Analyzing PBT and TPCs provides a clearer insight into how environmental factors affect whole-organism performance. However, data on how food availability impacts PBT and the shape of TPCs in reptiles is limited, with most research focusing on species adapted to tropical or subtropical climates. Previous studies on Chinese blue-tailed skinks (*P. chinensis*) (Shu et al. 2010), ornate tree lizard (*U. ornatus*) (Gilbert and Miles 2016) and southern African rock agama (*Agama atra*) (Van Berkel and Clusella-Trullas 2018) uncovered that food deprivation decreases the PBT of lizards. Additionally, Gilbert and Miles (2016) showed that the subtropical lizard, *U. ornatus* kept under suboptimal food condition had wider performance breadth, indicating a shift in the shape of thermal performance curves due to restricted food access.

The aim of our study was to investigate the effects of short-term starvation on the PBT and TPCs of a temperate-zone lizard species. The European green lizard (*Lacerta viridis*) serves as an ideal model system for examining how food deprivation impacts thermal biology as this species’ biology is well-known and it is widely used in physiological and behavioral studies (Horváth 2017; Mészáros et al. 2017, 2019) and due to its adaptability to temperate environments, contrasting with the more commonly studied tropical or subtropical species.

Building on the mentioned previous studies, we hypothesized that food deprivation would lower the preferred body temperature (i), thermal optimum (ii), and maximum performance (iii) of *Lacerta viridis*, while expanding the temperature range for effective performance (iv) under conditions of starvation.

## Material and methods

### Field sampling and housing

During the mating season of 2017, 40 male *L. viridis* individuals were collected in the vicinity of Tápiószentmárton, Hungary (47° 20′ 25″ N, 19° 47′ 11″ E, WGS84). Lizards were captured individually with a noose and then transferred to the laboratory at the Department of Systematic Zoology and Ecology, Eötvös Loránd University.

The snout-to-vent length (SVL) of each individual was measured using a digital caliper (Mitutoyo, Kawasaki, Japan) with a precision of 0.01 mm.

Lizards were housed individually in plastic terrariums (80 × 40 × 60 cm) equipped with sand substrate and a shelter. Water was provided ad libitum during the entire duration of their captivity. Each of the housing boxes was equipped with a 40 W spot lamps (OSRAM, Augsburg, Germany) suspended 20 cm above the substrate to maintain the optimal temperature range of 22.5–33.8 °C (Rismiller and Heldmaier 1988). A natural photoperiod of 14 h of light and 10 h of darkness was maintained throughout the whole experiment. Illumination was supplied by Repti Glo 2.0 Full Spectrum Terrarium Lamps (ExoTerra, Rolf C. Hagen Inc., Holm, Germany), which emitted minimal heat.

### Food treatment

28 male individuals were divided into two food treatment groups while the remaining 12 lizards were used to assess the CT_min_ and CT_max_ values (see below). Lizards in the optimal food treatment group (N = 14) were provided with 3 mealworms (*Tenebrio molitor*) per day, while those in the suboptimal treatment group (N = 14) received only 1 mealworm every two days. Individuals in the optimal treatment group could not consume all the mealworms, therefore we consider food availability in this group ad libitum. In contrast, the starved lizards consumed all the mealworms and actively searched for more, suggesting that the food amount was suboptimal for them. To induce short-term food deprivation, the food treatment began 10 days before the locomotor performance tests and was continued throughout the duration of the tests. However, performance tests were not conducted prior to the initiation of the food treatments. We selected a 10-day treatment period based on prior studies showing it is sufficient to induce measurable effects of short-term food deprivation on physiological performance while minimizing stress and avoiding irreversible impacts (McCue et al 2017). Body weight (BW) was measured before and after the 10-day food treatment period, using a digital scale (Ohaus Scout Pro SPU-2001; PineBrook, NJ) to the nearest 0.1 g.

There were no significant differences in body weight between the two groups before the treatments (Table S1). We examined the impact of food treatment on body weight using paired t-tests. In the optimal treatment group, there was a significant increase in body weight (Table S2; Figure S1), whereas in the suboptimal treatment group, body weight significantly decreased (Table S2; Figure S2).

### Preferred body temperature and thermal performance curves

We first determined the PBT of each lizard by placing them individually in a longitudinal terrarium (100 × 20 cm) with a temperature gradient, allowing for free thermoregulation. Prior to starting the measurements, we provided a 60-min acclimatization period to ensure the animals to adjust to the new environment. Following this period, body temperature was recorded every 2 min using a laser thermometer (Raytek Raynger ST, Raytek GmbH, Berlin, Germany) over a 60-min interval. Green lizards quickly acclimate to new environments, and we observed no signs of stress during measurements as we were careful and quiet to ensure the brief measurement period did not disturb their behavior. This methodology was adopted from previous studies that have effectively measured preferred body temperature (Gilbert and Miles 2016). PBT for each specimen was determined by calculating the average of the body temperature measurements.

Locomotor performance variables were assessed after food treatment using TPCs (Angilletta et al. 2002a, 2002b). To determine the x-intercepts of the TPCs, we identified the temperatures at which the animals displayed zero performance, denoted as critical thermal minimum (CT_min_) and critical thermal maximum (CT_max_). These temperatures were assessed on 12 lizards that were not part of the previously described experiment. For 6 lizards, body temperature was lowered using icepacks until they were unable to exhibit the righting reflex (performance reaching zero) (Spellerberg 1972; Voituron et al. 2002). For the other 6 lizards, body temperature was increased by warming under light bulbs until they were unable to show the righting reflex. A laser thermometer (Raytek Raynger ST, Raytek GmbH, Berlin, Germany) was employed to measure body temperature at the point of zero performance. Based on the average measurements, CT_min_ was recorded as 8.93 °C, while CT_max_ was determined to be 38.92 °C. Following this, we established two additional temperatures evenly distributed between CT_min_ and T_pref_, and between T_pref_ and CT_max_ (T1 = 20 °C; T2 = 25 °C; T_pref_ = 30 °C; T4 = 35 °C; T5 = 37 °C). This division resulted in a six-part temperature scale. The preferred temperature of the species (T_pref_) was defined as the average of the 28 individual’s PBTs.

We evaluated the locomotor performance of each lizard across the five previously designated temperature values (T1 = 20 °C; T2 = 25 °C; T_pref_ = 30 °C; T4 = 35 °C; T5 = 37 °C). Using the previously described methods to experimentally decrease or increase body temperature, we adjusted each individual to one of the five pre-set temperatures before placing them in a circular running arena with a 32 cm radius. We divided the arena into eight equal sections, each measuring 25.25 cm, resulting in a total perimeter of 202 cm. The arena was pre-heated or pre-cooled using light bulbs and ice packs, respectively, to match the lizards’ body temperature, ensuring a stable body temperature and preventing any fluctuations. The track temperature was frequently monitored with a laser thermometer throughout the trials to maintain consistency. The lizards were encouraged to run by hand, ensuring they reached their maximum speed until they no longer exhibited the righting reflex. This approach was used to elicit maximal sprint performance, which is crucial for accurately measuring TPCs. All of the performance tests were conducted by the same person. The measured distances were: T1 (99.3 ± 48.4 m, range: 30.3–200.0 m), T2 (133.0 ± 62.6 m, range: 46.5–333.0 m), T_pref_ (318.0 ± 108.0 m, range: 158.0–580.0 m), T4 (120.0 ± 44.9 m, range: 54.5–214.0 m) and T5 (114.0 ± 85.1 m, range: 32.3–416.0 m). The number of sections covered by each individual at each temperature was recorded and used in the subsequent analyses as the total distance run. Lizards ran only once a day, and between running days, a 1-day resting period was provided. Following all experiments, the lizards underwent a 2-day resting period and were released at the site of capture. No lizards experienced mortality or injuries as a result of handling, sampling, or treatments.

To generate individual TPCs, we plotted the performance of each trial against the corresponding body temperature and applied a Kumaraswamy function (Cordeiro and de Castro 2011; Jones 2009; Gómez Alés et al 2018; Mészáros et al 2019) for all individuals separately (Figure S3 and S4). From the resulting curves, we derived the thermal optimum (T_o_), the maximum performance (P_max_) and the performance breadth (B_80_) variables (Table 1). T_o_ indicates the peak point on the curve along the x-axis (the temperature value where performance is at its highest), while P_max_ is the peak point along the y-axis (the maximum performance). B_80_ represents the difference between the two temperature values where performance is at least 80% of the maximum performance (Angilletta et al. 2002b). Performance curves and performance variables were created in TableCurve 2D (SYSTAT Software Inc. 2002).

**Table 1:** Summary of thermal and morphologic variables in two treatment groups (optimal and suboptimal feeding conditions)

Variable	Mean ± SD		Min		Max	
	Opt	Sub	Opt	Sub	Opt	Sub
PBT (°C)	36.2 ± 0.79	35.6 ± 1.55	34.6	31.1	37.5	38.4
T_o_ (°C)	30.7 ± 0.98	31.5 ± 1.99	28.7	29.7	32.2	35.5
P_max_ (°C)	157.0 ± 65.6	141.0 ± 44.2	75.2	85.3	320.0	237.0
B_80_ (°C)	5.83 ± 1.07	7.18 ± 1.41	2.94	5.39	7.27	10.0

Mean ± SD (standard deviation), minimum (Min), and maximum (Max) values are presented for each variable across treatment groups. PBT (preferred body temperature) is the temperature range selected by individuals in a thermal gradient. To (thermal optimum) is the temperature at which the highest performance occurs, while Max indicates peak performance. B80 represents the temperature at which performance reaches at least 80% of the maximum. SVL denotes body length from snout to cloaca

### Statistical analyses

We examined the normality of both dependent and independent variables by visual inspection of histograms and q–q plots. Since our thermal performance variables did not follow normal distributions, we applied a Box–Cox transformation (Box and Cox 1964) to T_o_ and B_80_, log-transformed P_max_, and square root-transformed the PBT variable to achieve normalization. To address inter-correlation and collinearity, we carried out mass correlations which showed that none of the variables displayed strong correlations (R^2^ < 0.47). Dependent and independent variables were standardized to a mean of 0 and a standard deviation of 1 to improve model convergence.

To assess the impact of the food treatment on locomotor performance, we conducted four distinct General Linear Models (GLMs). In the three models PBT, T_o_, P_max_ and B_80_ were included separately as dependent variables while independent variables in all models encompassed food treatment and SVL.

Model simplification was assessed by the Akaike’s Information Criteria (Cavanaugh and Neath 2019). Alongside significance values, we also presented effect sizes as eta-squared (η^2^ = 0.01 is considered small, η^2^ = 0.06 is considered a medium effect size, and η^2^ > 0.14 is considered a large effect size) (Møller and Jennions 2002).

We performed model diagnostics on each model. Variance Inflation Factor analyses (Dormann et al. 2013) indicated the absence of collinearity in all models (VIF = 1.05). Homoscedasticity was assessed through visual inspection of diagnostic plots and Breusch–Pagan Tests (P > 0.608) (Breusch and Pagan 1979). Furthermore, all model residuals exhibited normal distribution as confirmed by visual inspection of histograms and q–q plots and Shapiro–Wilk tests (P > 0.152).

All analyses and the graphical presentation of the results were performed using the R software v. 4.2.2 and RStudio v. 2022.07.2 (R Core Team 2021).

## Results

**Fig. 1:**
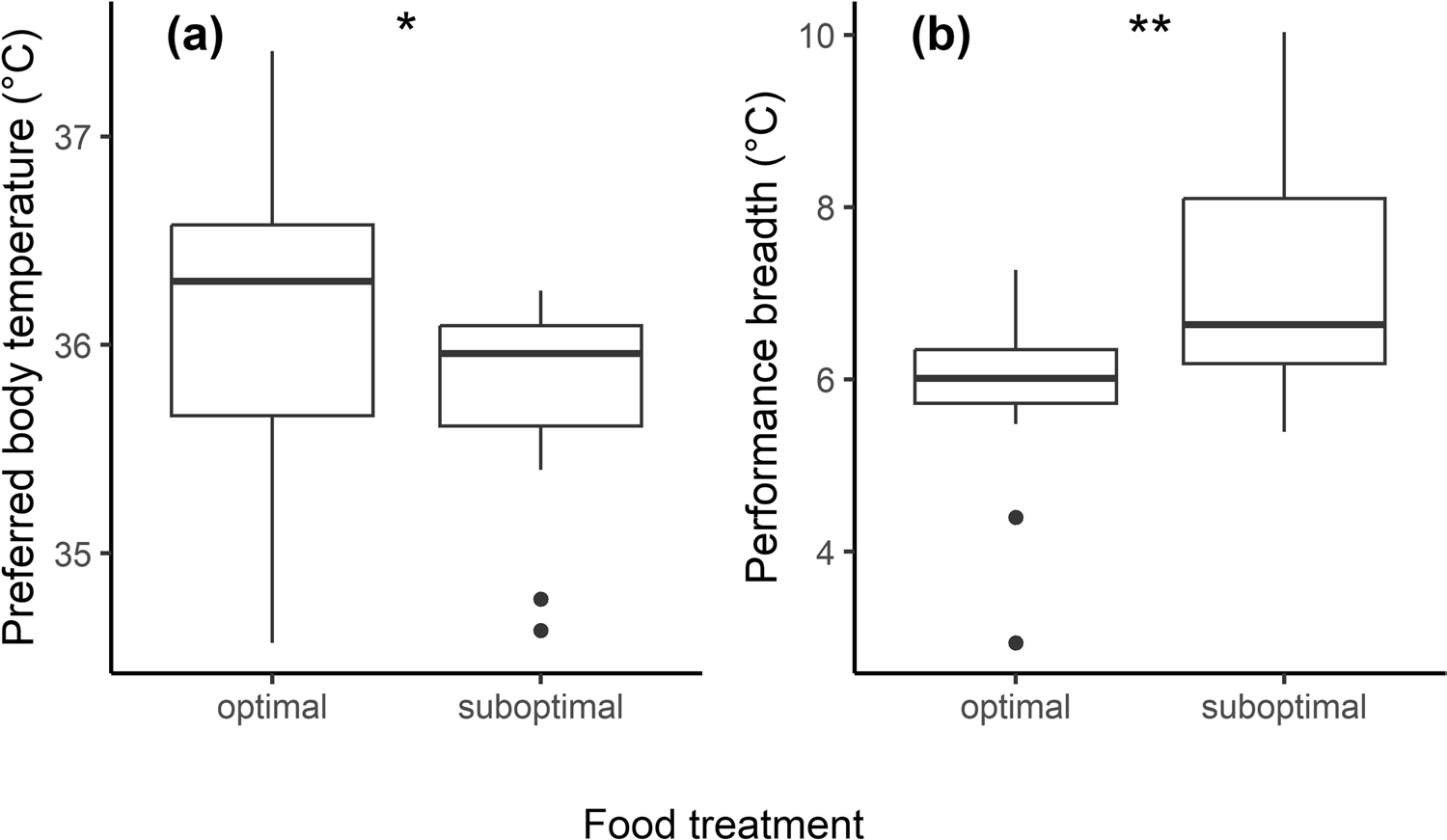
The difference of the preferred body temperature (**a**) and the performance breadth (**b**) of the male European green lizards (*Lacerta viridis*) in the optimal and suboptimal food treatment groups (median ± SD). Performance breadth or effective performance is the temperature range (°C) where performance is at least 80% of the maximum performance

The GLM examining the food deprivation on PBT showed a significant influence of food treatment (Table 2). Lizards subjected to the suboptimal food treatment showed decreased preferred body temperatures, while lizard under optimal food conditions preferred higher temperatures (Fig. 1a).

**Table 2:** The results of the three General Linear Models with individual preferred body temperature (PBT), temperature of maximum performance (T_o_), maximum performance (P_max_) and performance breadth (B_80_) as response variables and food treatment, snout-vent-length (SVL) as independent variables

Response variable	Predictors	β	sd	t	p	η^2^	AIC
PBT	Food treatment	**0.485**	**0.232**	**2.092**	**0.048**	**0.154**	**49.45**
	SVL	−0.174	0.114	−1.525	0.141	0.082	49.45
T_o_	Food treatment	0.105	0.294	0.359	0.723	0.005	62.53
	SVL	0.010	0.151	0.069	0.946	0.001	64.53
P_max_	Food treatment	−0.356	0.379	−0.949	0.352	0.032	83.05
	SVL	0.287	0.188	1.526	0.139	0.082	82.04
B_80_	Food treatment	**0.954**	**0.337**	**2.833**	**0.009**	**0.236**	**76.91**
	SVL	−0.116	0.178	−0.655	0.519	0.013	78.43

Non-significant results are shown as seen at the time of removal during the model selection based on AIC values. Effect sizes are indicated as η^2^, and significant effects are highlighted in bold font

Additionally, our results showed that food treatment had a significant influence on the performance breadth variable (B_80_) (Table 2). Lizards subjected to suboptimal food conditions displayed a broader performance breadth, indicating their ability to sustain 80% of maximum performance across a wider temperature range. Conversely, individuals under optimal food conditions exhibited effective performance within a narrower temperature range (Fig. 1b).

P_max_ and T_o_ did not exhibit correlations with any of the explanatory variables (Table 2).

## Discussion

Performance is shaped through the interaction of temperature with several environmental and physiological factors, while the TPC is a product of these interconnected elements (Litchman and Thomas 2023). Fluctuating food resources have been described as ecological stressors capable of affecting physiology, behavior and locomotor performance, altering PBT and the shape of the TPCs in tropical and subtropical lizards and ultimately affecting survival and reproductive success (Lailvaux et al. 2018; Litchman and Thomas 2023; Orton et al. 2020). In order to understand the cumulative impact of food resources on locomotor performance of a temperate lizard, our study sought to investigate how starvation influence PBTs and TPCs of *L. viridis*. Our short-term food deprivation treatment lowered the preferred body temperature of the lizards and expanded their effective performance range compared to the well-fed treatment group, indicating that food availability is a crucial environmental stress factor shaping the thermal performance of a temperate lizard species. However, we did not observe any effects of food treatment on the thermal optimum or maximum performance of the lizards.

Our initial result indicated that lizards with adequate food resources selected higher body temperatures (Fig. 1a), in contrast to those experiencing food deprivation. Multiple studies have shown that reptiles increase their body temperature after feeding to support higher metabolic rates necessary for digestion (Huey 1982; Andrade et al. 2005; Secor 2009). Available food and the ability to reach higher body temperatures can support growth in lizards by enabling them to stay near optimal activity temperatures, which extends foraging time and boosts digestion rates (Huey 1982; Huey and Kingsolver 2019). Consistent with findings from other studies (Shu et al. 2010; Gilbert and Miles 2016; Van Berkel and Clusella-Trullas et al. 2011; Padilla Perez et al. 2021), we also demonstrated that food deprivation lowered the preferred body temperature of lizards, indicating that lizards with limited food resources respond by reducing their body temperature (Fig. 1a). As a potential explanation, it is plausible that in order to cope with malnutrition, individuals sustain a compromised body temperature. Lizards might adopt a behavioral strategy to survive periods of reduced food availability by lowering their body temperature to minimize energy expenditure (Huey 1982). Ectotherms have the capacity to sustain a body temperature considerably lower than the optimum (Van Berkel and Clusella-Trullas 2018) which is minimally essential and adequate for foraging and survival. To achieve this lower body temperature, lizards engage in thermoregulation by seeking cooler patches. This strategy enables ectothermic organisms to maintain a significantly reduced but still sufficient level of performance during periods of starvation. Unfortunately, the warming of thermal habitats by human-induced rapid environmental changes (HIERC) like urbanization, habitat degradation, fragmentation and climate change may diminish the availability of these cooler sites, leading to unsuccessful adaptations by lizards to their changing environment (Huey and Kingsolver 2019; Sinervo et al. 2010). This loss could restrict ectotherms to shorter activity periods, potentially resulting in further declines in food intake. This phenomenon is referred to as a “metabolic meltdown”, due to the simultaneous factors of declining energy intake, increasing metabolic costs at higher temperatures, and the limitations on activity imposed by warming conditions (Huey and Kingsolver 2019).

Our study also found that food availability influenced the temperature range in which lizards can maintain effective performance. These results indicate that lizards with optimal food resources maintained effective performance within a narrower temperature range, whereas periods of fasting forced them to sustain effective performance across a broader temperature range (Fig. 1b). This observation is consistent with the findings of Gilbert and Miles (2016) in *U. ornatus* lizards.

Two main hypotheses guide the study of thermal performance curve evolution: the “warmer is better” hypothesis and the “Jack-of-all-temperatures is a master of none.” The “warmer is better” hypothesis suggests that organisms with high optimal temperatures achieve higher maximal performance due to the thermodynamics of biochemical systems (Huey & Kingsolver 1989). In contrast, the “Jack-of-all-temperatures” hypothesis proposes a trade-off between peak performance and performance range, based on the balance between enzyme flexibility and stability. This theory predicts that specialist phenotypes optimize their performance in stable environments, whereas generalist phenotypes maintain near-maximum performance in variable environments by expanding the temperature range of effective performance. According to this theory, lizards that can achieve high performance within a narrow temperature range are considered thermal specialists, while those that perform well across a broad temperature range are classified as thermal generalists (Huey & Kingsolver 1989). Our results indicate that food deprivation has resulted in the expansion of performance breadths which may be a physiological strategy for thermal generalists to sustain similar performance levels over a broader temperature range during food shortages, thus minimizing stress when food is scarce.

We acknowledge the absence of pre-experimental data for direct comparison with post-experimental measurements for each individual. However, we believe that the results remain mainly comparable as no significant differences in body weight were observed between the two groups prior to treatment. Furthermore, the optimal treatment group demonstrated weight gain, while the suboptimal treatment group exhibited weight loss. Given that body weight is a reliable indicator of nutritional status, we assumed the lizards were probably in similar condition before our experiments. While this assumption supports the comparability of our results, it is important to interpret these findings in the context of the lack of pre-experimental data.

In conclusion, we showed that food availability, a crucial environmental stressor, plays a significant role in shaping the thermoregulation strategy of an ectothermic organism by influencing the preferred body temperature and the thermal performance curves of male *L. viridis*. This form of plasticity in thermoregulation behavior could secure the capability to manage the consequences of environmental changes and adjust to new habitats. However, their adaptability might be confined within genetic constraints, potentially insufficient to cope with rapid and prolonged changes, such as global warming (Forster et al. 2023), habitat loss and urbanization (Hoffmann and Sgrò 2011). The increasing effects of climate change and human-induced habitat modifications are expected to result in more frequent and severe climatic and weather conditions Cook et al. 2018). This may introduce new and intensified environmental stressors to populations and species (Sinervo et al. 2010). It is therefore essential to assess the impact of the ongoing and the potentially occurring changes on species and populations in order to better inform conservation policies/effort and determine the need for human intervention.

## Supplementary Information

Below is the link to the electronic supplementary material.

Supplementary file1 (DOCX 1033 kb)

## Data Availability

The datasets used during the current study will be available after acceptance in the ARP Research Data Repository: https://hdl.handle.net/21.15109/ARP/B5GITE
