# Supplementary material for: Impact of food availability on the thermal performance curves of male European green lizards (Lacerta viridis)
Source: Oecologia. 2025 Apr 1;207(4):57. doi: 10.1007/s00442-025-05699-z (PMC11961467; doi:10.1007/s00442-025-05699-z)
Supplement: Supplementary file 1 — Supplementary file1 (DOCX 1033 kb) [file 442_2025_5699_MOESM1_ESM.docx]

**Title:** Impact of food availability on the thermal performance curves of male European green lizards (*Lacerta viridis*)

**Journal:** Oecologia

**Authors:**

Boglárka Mészáros^1, 2 *^

Lilla Jordán^2, 3^

Orsolya Molnár^2^

János Török^2, 4^

^1^HUN-REN Balaton Limnological Research Institute, Klebelsberg Kuno Street 3., H-8237 Tihany, Hungary

^2^Behavioural Ecology Group, Department of Systematic Zoology and Ecology, ELTE Eötvös Loránd University, Pázmány Péter sétány 1/C, 1117 Budapest, Hungary

^3^Leibniz Institute for Zoo and Wildlife Research, Alfred-Kowalke-Str. 17, 10315 Berlin, Germany

^4^HUN-REN-ELTE-MTM Integrative Ecology Research Group, 1117 Budapest, Pázmány Péter sétány 1/C

*corresponding author: Boglárka Mészáros

e-mail: [meszaros.boglarka@blki.hun-ren.hu](mailto:meszaros.boglarka@blki.hun-ren.hu)

1. **Supplementary Tables**

**Table S1.** Summary of independent t-test results comparing body weight between optimal and suboptimal conditions prior to the food treatment. A p-value below 0.05 would indicate a statistically significant difference between the two groups

|  | Optimal mean | Suboptimal mean | t-value | df | p-value |
| --- | --- | --- | --- | --- | --- |
| Body weight | 18.14 | 20.13 | -1.15 | 24 | 0.26 |

**Table S2.** Results of paired t-tests comparing mean values of body weight before and after treatment for each food treatment group. A p-value below 0.05 indicates a statistically significant change in body weight within the treatment group.

| Food treatment | Before mean | After mean | t-value | df | p-value |
| --- | --- | --- | --- | --- | --- |
| Optimal | 18.14 | 18.97 | -2.17 | 13 | 0.05 |
| Suboptimal | 20.13 | 18.61 | 4.49 | 13 | < 0.001 |

1. **Supplementary Figures**

**Figure S1**: Boxplots showing the body weight (g) of lizards before and after the optimal food treatment. The asterisk (*) above the boxplots indicates a statistically significant difference (p ≤ 0.05) in body weight between the two time points.


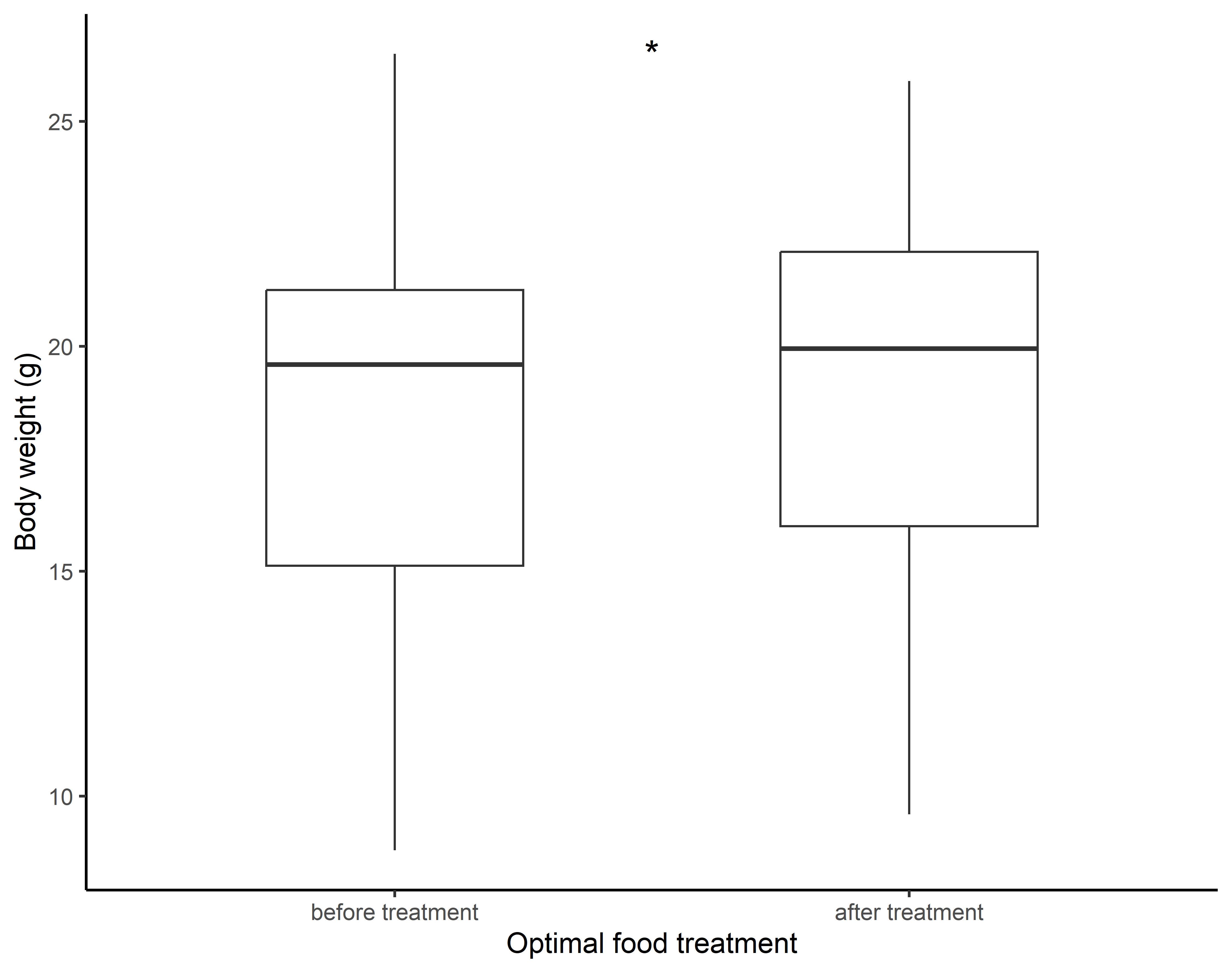


**Figure S2**: Boxplots showing the body weight (g) of lizards before and after the suboptimal food treatment. The three asterisks (***) above the boxplots indicate a highly significant difference (p < 0.001) in body weight between the two time points.


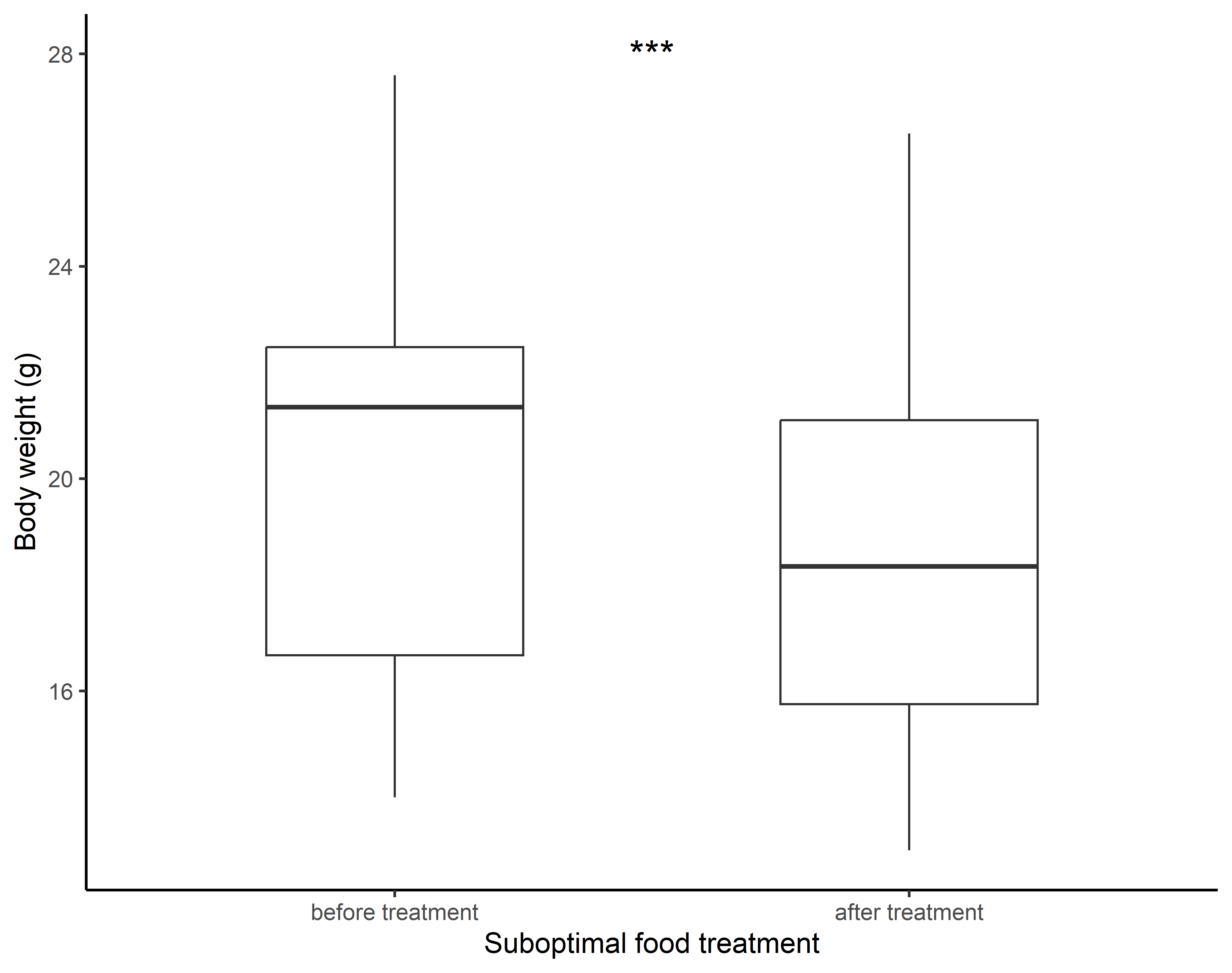


**Figure S3**: Figures showing the individual thermal performance curves (TPCs) for male European green lizards (*Lacerta viridis*) in the optimal food treatment group, generated using the Kumaraswamy function in TableCurve 2D. The y-axis (dist) represents locomotor performance, defined as the maximum sections (1 section = 25.25 cm) run during the trials at each temperature (blue dots), while the x-axis (temp) displays the five temperatures at which performance was measured (T1 = 20 °C; T2 = 25 °C; Tpref = 30 °C; T4 = 35 °C; T5 = 37 °C) and CT_min_ (8.93 °C) and CT_max_ (38.92 °C). Each curve is labeled with the individual ID

| 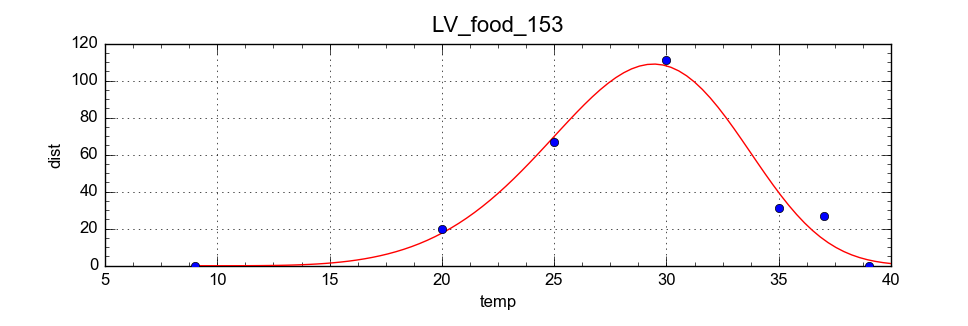 |
| --- |
| 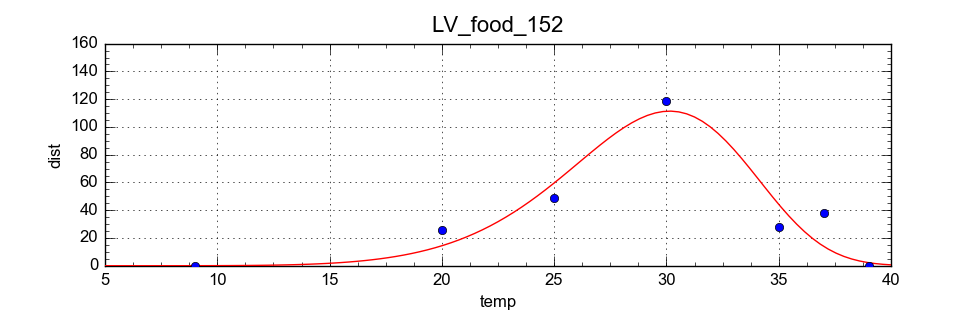 |
| 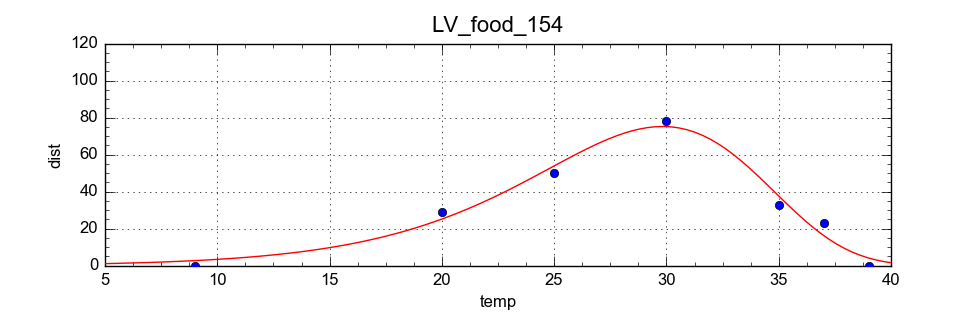 |
| 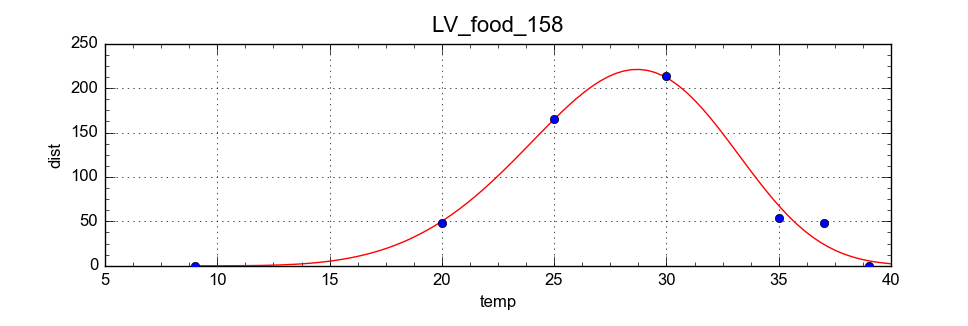 |
| 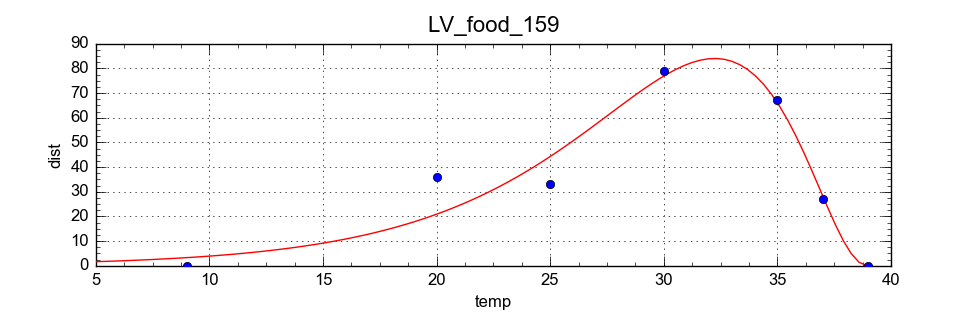 |
| 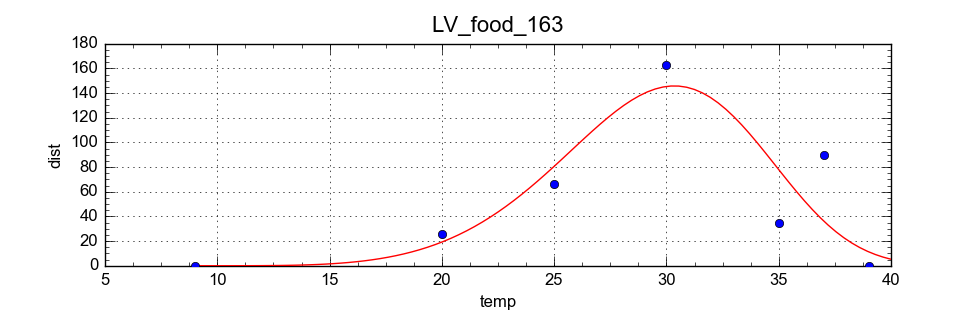 |
| 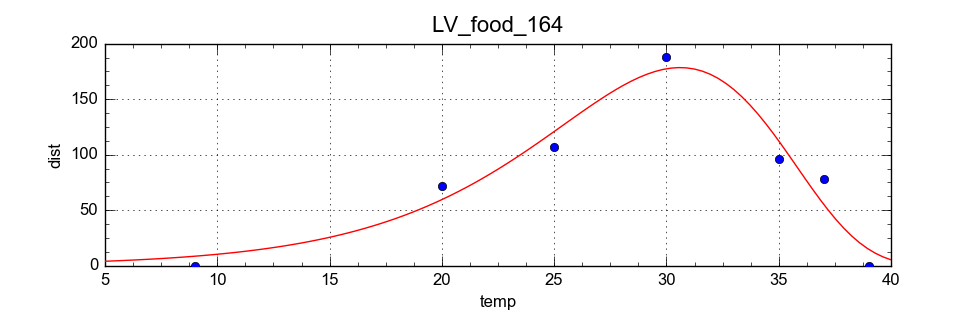 |
| 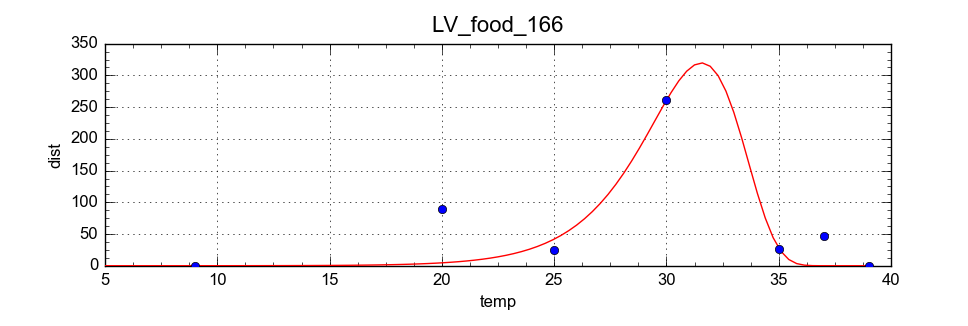 |
| 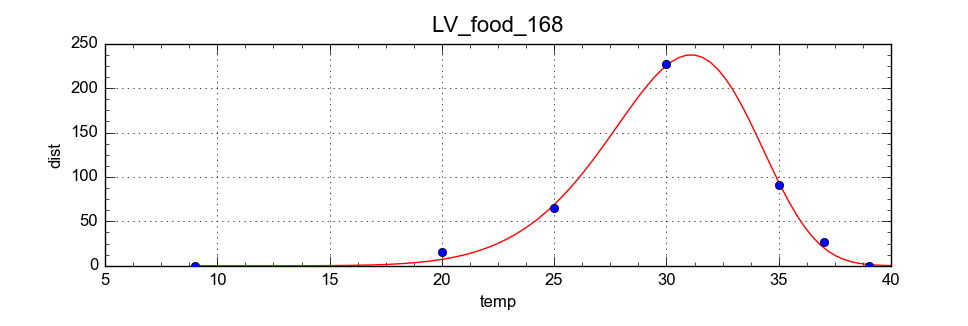 |
| 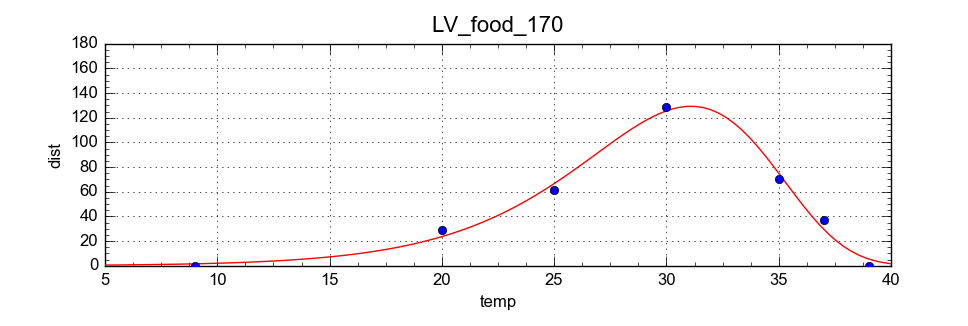 |
| 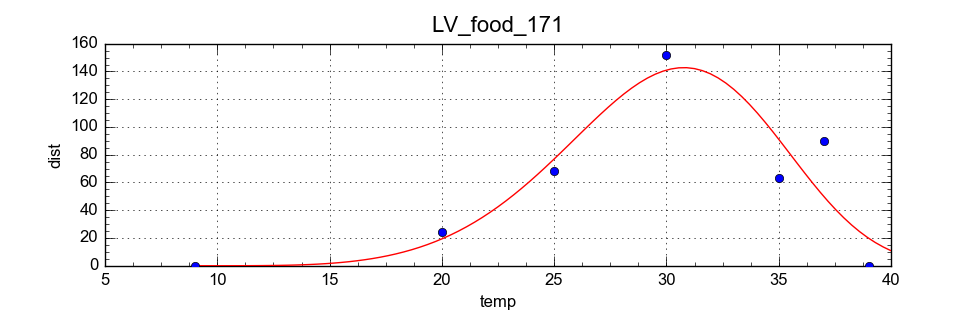 |
| 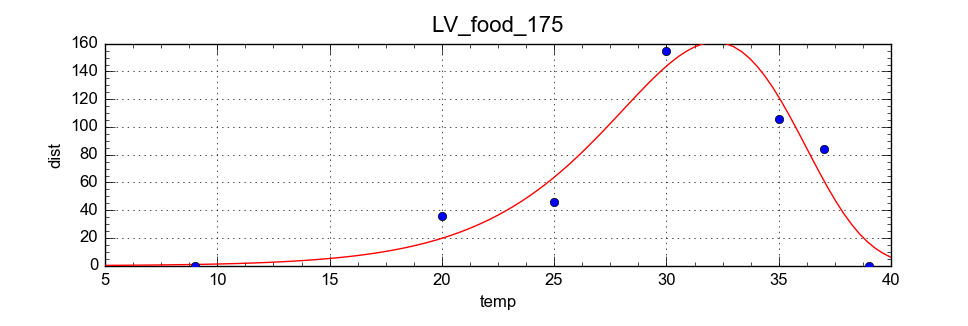 |
| 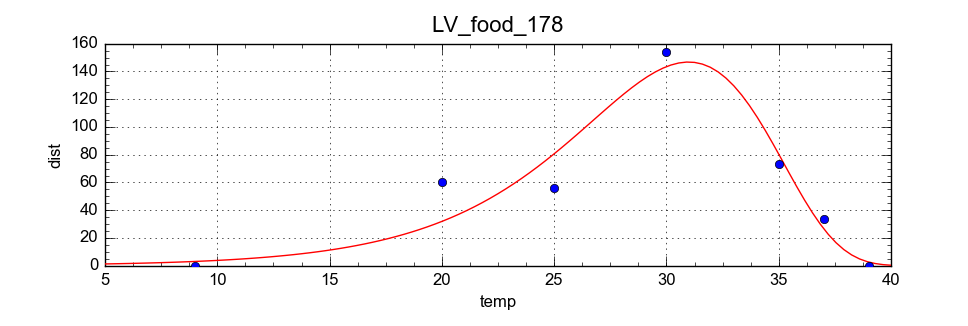 |
| 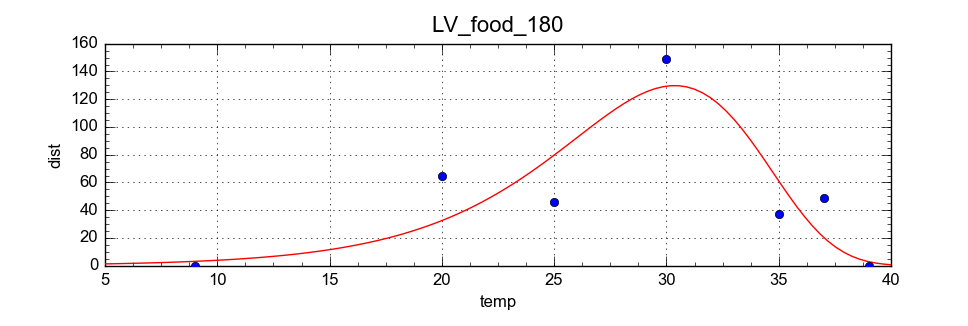 |

**Figure S4**: Figures showing the individual thermal performance curves (TPCs) for male European green lizards (*Lacerta viridis*) in the suboptimal food treatment group, generated using the Kumaraswamy function in TableCurve 2D. The y-axis (dist) represents locomotor performance, defined as the maximum sections (1 section = 25.25 cm) run during the trials at each temperature (blue dots), while the x-axis (temp) displays the five temperatures at which performance was measured (T1 = 20 °C; T2 = 25 °C; Tpref = 30 °C; T4 = 35 °C; T5 = 37 °C) and CT_min_ (8.93 °C) and CT_max_ (38.92 °C). Each curve is labeled with the individual ID

| 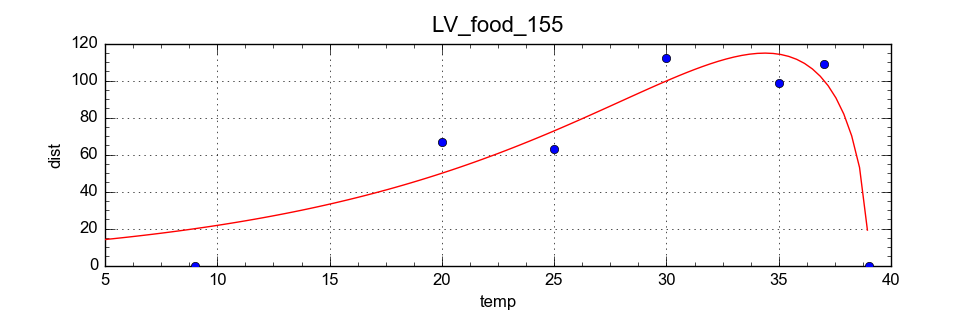 |
| --- |
| 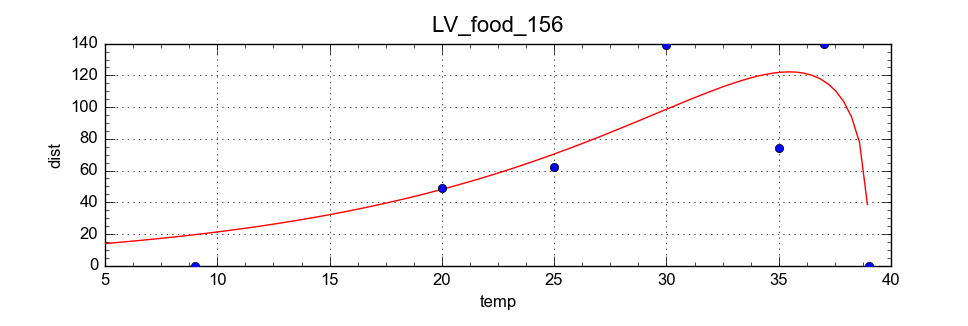 |
| 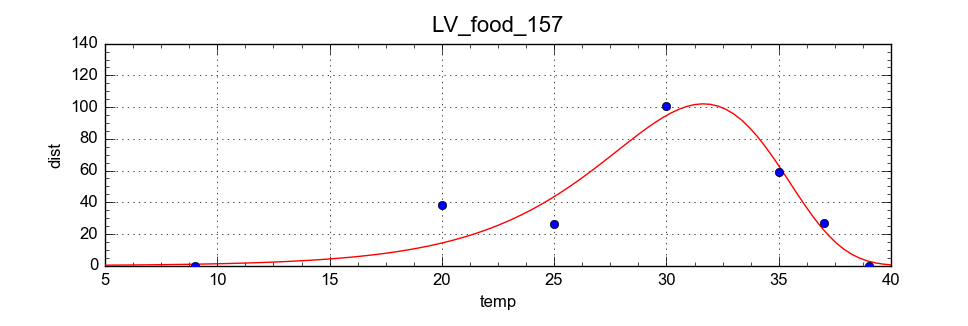 |
| 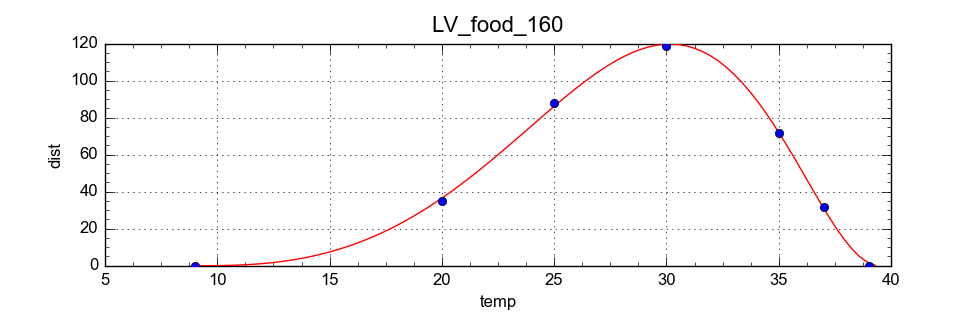 |
| 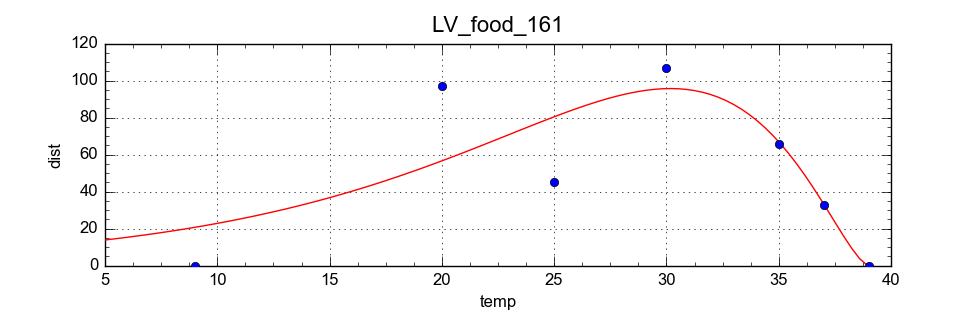 |
| 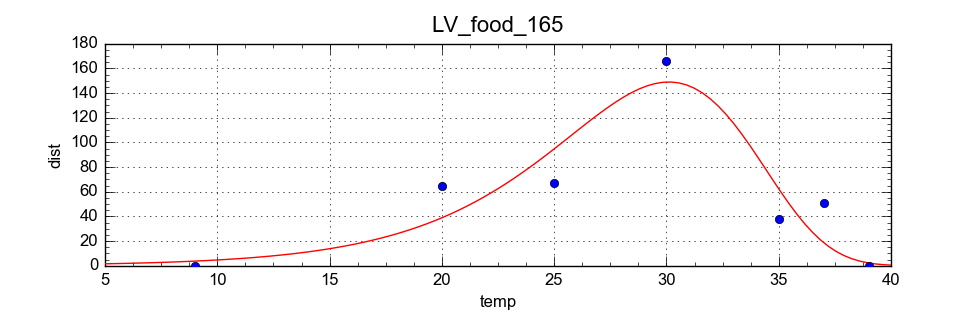 |
| 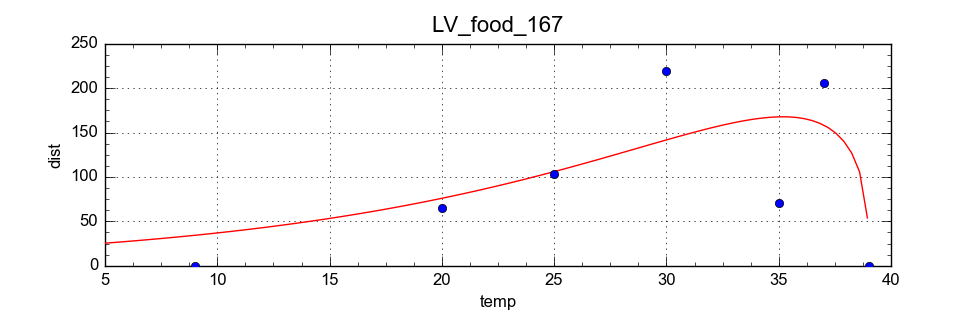 |
| 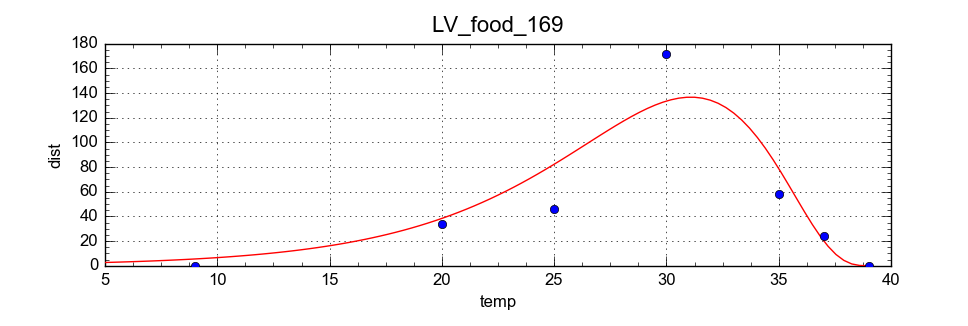 |
| 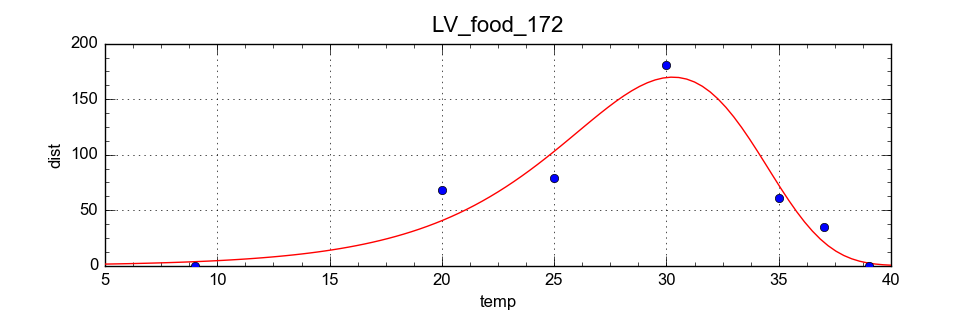 |
| 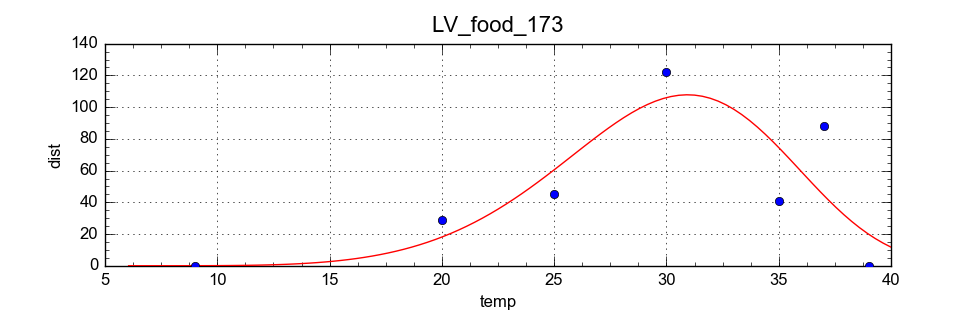 |
| 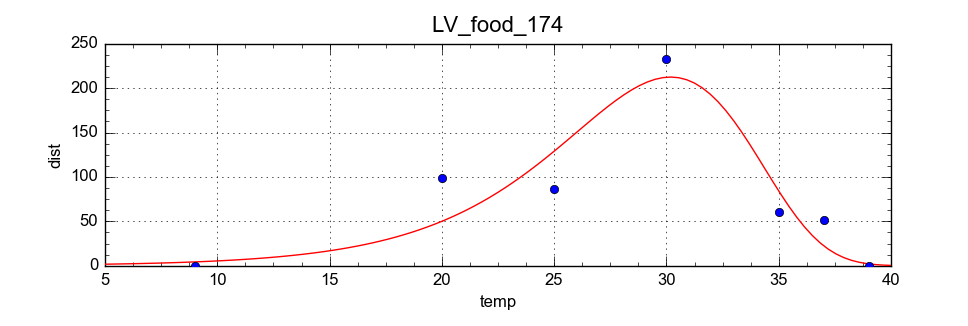 |
| 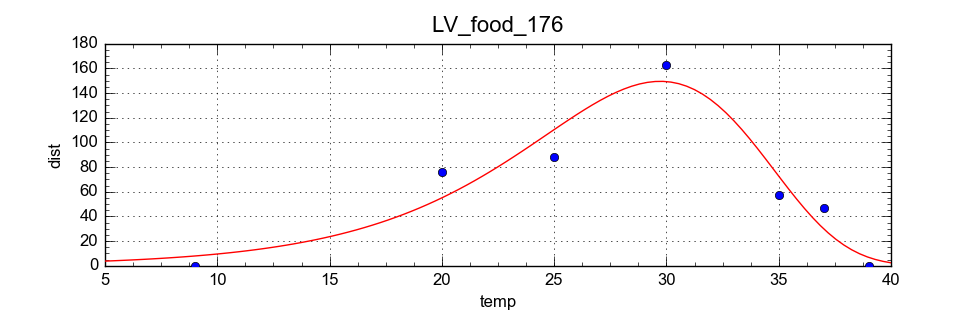 |
| 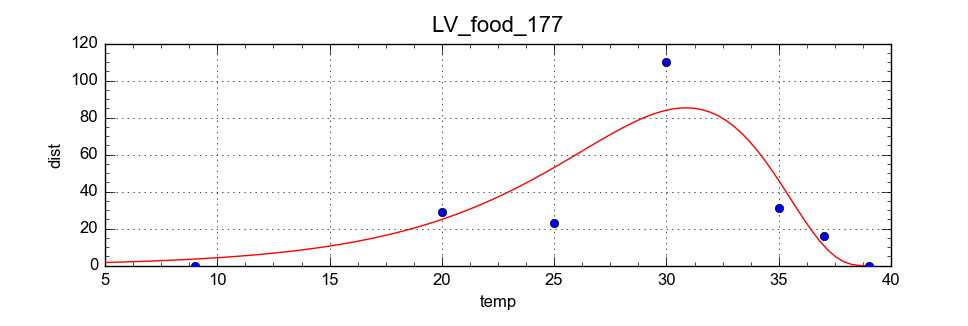 |
| 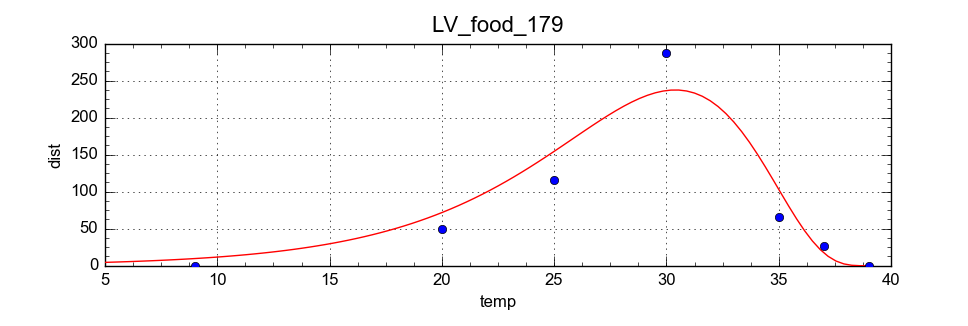 |
